# Association between metabolic dysfunction-associated fatty liver disease and cardiovascular autonomic neuropathy in type 2 diabetes

**DOI:** 10.3389/fendo.2025.1711660

**Published:** 2026-01-28

**Authors:** Rong Peng, Yaoyun Ao, Enni Cen, Junnian Chen, Zeshan Huang, Xiaoying Fu, Jian Kuang, Shuiqing Lai, Shuting Zhang

**Affiliations:** 1 Department of Endocrinology, Guangdong Provincial People’s Hospital(Guangdong Academy of Medical Sciences), Southern Medical University, Guangzhou, Guangdong, China; 2 Department of Endocrinology, The People’s Hospital of JiangMen, JiangMen, Guangdong, China; 3 Department of Gastroenterology, The People’s Hospital Medical Group of Xiangzhou, Zhuhai, Guangdong, China

**Keywords:** cardiovascular autonomic neuropathy, cardiovascular autonomic reflex tests, FIB-4 index, metabolic dysfunction-associated fatty liver disease, type 2 diabetes mellitus

## Abstract

**Objective:**

This cross-sectional study aimed to elucidate the relationship between metabolic dysfunction-associated fatty liver disease (MAFLD) and diabetic cardiovascular autonomic neuropathy (DCAN) in patients with type 2 diabetes mellitus (T2DM).

**Methods:**

The study involved patients with T2DM. DCAN was diagnosed using standardized cardiovascular autonomic reflex tests (CARTs) with a total score≥2. MAFLD was defined by the presence of fatty liver disease and T2DM, excluding other liver diseases. The fibrosis-4 (FIB-4) >1.3 indicated a potential risk of fibrosis based on prior studies.

**Results:**

Overall, 30.52% (76/249) patients had DCAN. Patients with MAFLD had a significantly higher prevalence of DCAN than those without (36.49% vs. 21.78%, *P=0.013*). Univariable analysis revealed a significant association between MAFLD and DCAN (OR = 2.06, 95% CI: 1.16-3.68, *P=0.014*). This association remained significant even after multivariable adjustment for demographics, diabetes duration, comorbidities (hypertension, diabetic peripheral neuropathy, diabetic retinopathy, metabolic syndrome), and renal function (adjusted OR = 2.76, 95% CI: 1.44-5.29, *P=0.002*). Among T2DM patients with MAFLD, a high FIB-4 index (>1.3) was independently associated with a substantially increased DCAN risk (adjusted OR = 2.81, 95% CI: 1.19-6.63, *P=0.018*).

**Conclusion:**

MAFLD is independently associated with a higher prevalence of DCAN in patients with T2DM. The risk was further amplified when high FIB-4 index (FIB-4 >1.3) was present among those with MAFLD. Hence, screening for MAFLD and its associated high FIB-4 levels may help identify patients with T2DM at a higher risk of DCAN.

## 1 Introduction

Diabetic cardiovascular autonomic neuropathy (DCAN) is a severe chronic diabetic complication that damages the autonomic fibers of the heart. Reportedly, 29 ~ 54% of patients with type 1 diabetes and 12 ~ 73% of patients with type 2 diabetes mellitus (T2DM) develop DCAN (1). The prevalence of definite DCAN in patients with T2DM is 15.3%, whereas that in patients with type 1 diabetes is 54% (2, 3). DCAN is easily overlooked as it has an insidious onset subtle symptoms. Severe clinical symptoms, such as orthostatic hypotension, silent myocardial infarction, and even sudden cardiac death, emerge only with the progress of nervous system damage (4). A prospective cohort analysis revealed a 1.9‐fold greater risk of silent myocardial infarction in participants with DCAN than those without (5). Among several tests used to diagnose DCAN, cardiovascular autonomic reflex tests (CARTs) have been recommended as the gold standard for DCAN, according to various guidelines and consensus statements (6–9). However, due to the absence of an automatic test system, the completion of CARTs and interpretation of results still rely on manual effort. Therefore, we developed an intelligent device for diagnosing CAN and verified its accuracy and reproducibility (10). In fact, there are few studies discussing the associations between CAN and other diseases.

Metabolic dysfunction-associated fatty liver disease (MAFLD), formerly referred to as non-alcoholic fatty liver disease (NAFLD), is one of the most common chronic liver diseases, with an estimated prevalence of 25% worldwide (11). As a liver disorder strongly linked to dysmetabolic conditions, diabetes, obesity, and metabolic syndrome are believed to be the most critical risk factors for MAFLD (12, 13). The current widespread prevalence of diabetes and obesity among the global population has also increased the prevalence of MAFLD. As reported in a meta-analysis, the pooled global prevalence of T2DM in patients with MAFLD is as high as 26.2% (14). The interaction between MAFLD and metabolic diseases also leads to an increase in cardiovascular diseases, the leading causes of mortality in MAFLD (15). Among patients with T2DM, those with NAFLD had a remarkably higher prevalence of coronary, cerebrovascular, and peripheral vascular diseases than those without NAFLD. This further highlights that NAFLD as a predictor of cardiovascular events (16). However, abnormal cardiac function, such as the presence of a left ventricular mass, has also been linked to advanced liver fibrosis among patients with NAFLD (17).

Despite these cardiovascular connections, the specific link between MAFLD and DCAN remains mechanistically plausible but clinically unknown. Pathophysiologically, MAFLD-driven systemic inflammation (elevated TNF-α and IL-6) and insulin resistance may predispose individuals to autonomic nerve injury by promoting endothelial dysfunction, oxidative stress in the vasa nervorum, and sympathetic overactivity (18, 19). However, the clinical evidence for this association is inconsistent. Some studies have suggested a link between hepatic steatosis and cardiac autonomic dysfunction in patients with and without T2DM (20). While MAFLD has been linked to an elevated risk of cardiovascular disease in patients with T2DM, its potential contribution to the development of DCAN risk among such patients remains understudied. Thus, the present study aimed to determine the prevalence of DCAN among T2DM patients with or without MAFLD and to further investigate the relationship between MAFLD and DCAN in patients with T2DM.

## 2 Materials and methods

### 2.1 Study population

**Figure 1:**
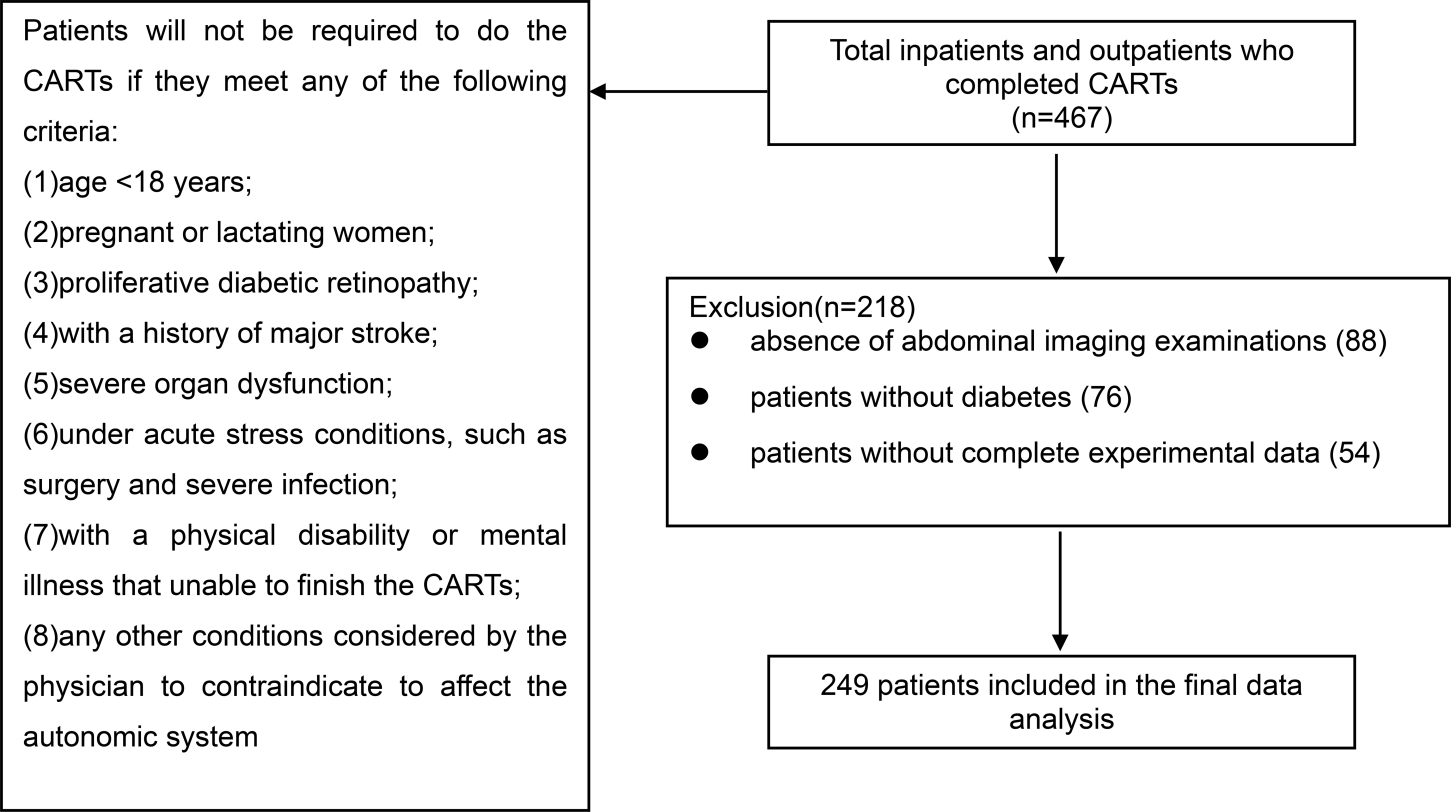
Study flow chart.

A cross-sectional study was conducted at the Department of Endocrinology of the Guangdong Provincial People’s Hospital between December 2021 and June 2025. The participants were diagnosed with T2DM according to the 1999 World Health Organization diagnosis criteria and the 2025 classification of diabetes mellitus (21, 22). Patients without contraindications proceeded to complete CARTs. Between 2021 and 2025, 467 participants who successfully completed the CARTs were initially enrolled. Following eligibility screening, 249 T2DM patients with complete baseline clinical data were included in the final analysis (exclusion criteria: 88 without abdominal imaging, 76 without a diagnosis of diabetes, and 54 with incomplete baseline data) (**Figure 1**). This study was approved by the Ethics Committee of Guangdong Provincial People’s Hospital. All the participants provided written informed consent.

### 2.2 Evaluation of MAFLD and liver fibrosis evaluation

MAFLD was defined as fatty liver present with overweight (BMI ≥ 23 kg/m^2^ in Asians), T2DM, or at least two metabolic risk factors according to the international expert consensus statement (23). For this study, which exclusively enrolled patients with type 2 diabetes mellitus (T2DM), MAFLD was diagnosed based on fatty liver plus the presence of T2DM, as T2DM alone constitutes a sufficient metabolic criterion within the consensus definition. To ensure diagnostic consistency in this study, participants meeting the “positive” criteria of MAFLD as mentioned above were further screened to exclude those with alcohol-associated fatty liver disease, viral infections (HBV, HCV, or HIV), drug-induced liver injury, or autoimmune hepatitis (24). The fibrosis-4 index (FIB-4) is a high performance, non-invasive scoring system used to predict the risk of liver fibrosis. As described in previous studies, participants were further grouped according to FIB-4 index (FIB-4 > 1.3 and FIB-4 ≤ 1.3) (25–29).

### 2.3 Evaluation of cardiovascular autonomic neuropathy

Cardiovascular autonomic neuropathy was assessed using CARTs. The CARTs were conducted using four standardized maneuvers, which generated five indices to evaluate the function of the sympathetic and the parasympathetic nerves. Cardiovascular parasympathetic function was evaluated using heart rate response to deep breathing (E/I test), orthostatic change (30:15 test), and Valsalva maneuver (Valsalva ratio). Cardiovascular sympathetic function was assessed by measuring the blood pressure response to orthostatic changes (orthostatic hypotension test) and sustained handgrip. Changes in the heart rate and blood pressure are critical indicators of CAN. Each maneuver was scored as 0 for normal, 0.5 for borderline, and 1 for abnormal (**Supplementary Table 1**) (30). CAN was defined as a total score of ≥ 2, while DCAN was defined as CAN (+) with a history of T2DM (31–33). The severity of CAN was further stratified into early CAN (one abnormal cardiovagal test result), definite CAN (at least two abnormal cardiovagal tests results), severe CAN (plus one or both of the blood pressure tests abnormal, or both borderline on the basis of definite CAN), and atypical CAN (any other combination of abnormal tests) (30). Conventional CARTs were performed by two trained physicians using common equipment, including a syringe connected to a manometer, dynamometer, double-headed stethoscope, mercury sphygmomanometer, and an ECG machine. In terms of time efficiency, reduced personnel requirements, and accuracy of measurements, a novel cardiovascular autonomic device was used to diagnose CAN (Cardiovascular Autonomic Nervous Function Multi Parameter Evaluation System Model R6000, REEM (Shenzhen) Healthcare Co., Ltd., Shenzhen, China). The device features a wireless respiratory flow sensor and a digital dynamometer, which transmit parameters during maneuvers, including deep breathing, the Valsalva maneuver, and sustained hand grip, thereby facilitating human–machine interaction. Its excellent accuracy and reproducibility were verified in comparison with conventional CARTs in our previous study (10).

To minimize potential influences on autonomic measurements, participants adhered to the following protocol: abstinence from strenuous exercise for 24h prior to testing; and, within the 2h before testing, fasting or consumption of a light meal, as well as avoidance of smoking, alcohol, coffee, and tea. During the tests, participants were asked to remain quiet and awake. Consistent with previous studies, the heart rate was continuously monitored during the examination, while blood pressure was measured at 1-min intervals (30, 34). A wireless ECG patch continuously collects ECG signals, whereas the blood pressure module intermittently measures blood pressure at prespecified intervals. In addition, the device automatically records action parameters, including air pressure during the Valsalva maneuver, deep breathing, duration of the exhalation and inhalation phases, and grip strength. It can automatically mark key time points associated with maneuvers, identify and process ECG artifacts, and generate a diagnosis upon completion of the examination.

### 2.4 Diabetic complications and comorbidities assessment

Diabetic peripheral neuropathy (DPN) was diagnosed based on the 2020 Guideline for T2DM Prevention and Treatment (35). Diabetic retinopathy (DR) was diagnosed using a series of ophthalmic tests performed by an experienced ophthalmologist before CARTs. Hypertension was defined as SBP/DBP>140/90 mmHg or use of antihypertensive medications. Participants with one or more of the specified criteria were considered to have dyslipidemia: serum total cholesterol ≥ 6.2 mmol/L, serum triglycerides ≥ 2.3 mmol/L, low-density lipoprotein cholesterol ≥ 4.1 mmol/L, or high-density lipoprotein cholesterol < 1.0mmol/L, or self-reported use of lipid-lowering medications (36). Metabolic syndrome was defined in accordance with the 2005 Statement for Diagnosis and Management of the Metabolic Syndrome (37). Obesity was defined as the BMI ≥ 28 kg/m^2^. Hyperuricemia was defined as an elevated level of uric acid (> 420μmol/L in men and > 360μmol/L in women).

### 2.5 Statistical analysis

All analyses were performed using R (version 4.2.3). Continuous variables are summarized as the mean ± standard deviation or medians [interquartile ranges]. Comparisons between groups were performed using Student’s t test, Mann–Whitney U test, or chi-square/Fisher’s exact tests as appropriate. Furthermore, univariate logistic regression analyses were performed to evaluate the correlation between clinical variables and DCAN. Multivariate logistic regression analyses were used to test for independent associations between the covariates and DCAN. Statistical significance was established with a two-sided *P*-value < 0.05.

## 3 Results

### 3.1 Demographic characteristics

A cross-sectional sample of 249 T2DM patients was enrolled in our study (**Figure 1**). The baseline demographic characteristics of the included and excluded groups were comparable, as detailed in **Supplementary Table 2**. The median age was 53 (41, 61) years, with a mean diabetes duration of 60 (10, 120) months. The average BMI was 25.81 ± 4.00 kg/m^2^ and the incidence of obesity was 29.32% (73/249). Among the 249 T2DM patients, the incidence rate of DCAN was 30.52% (76/249) (**Table 1**).

**Table 1:** Characteristics of T2DM patients with or without MAFLD.

	Total (n = 249)	MAFLD (n = 148)	non-MAFLD (n = 101)	*P* value
Male, n (%)	158 (63.45)	95 (64.19)	63 (62.38)	0.771
Age (years)	53 (41, 61)	52 (41, 59)	54 (43, 62)	0.211
Smoking, n (%)	74 (29.72)	45 (30.41)	29 (28.71)	0.774
Drinking, n (%)	34 (13.65)	25 (16.89)	9 (8.91)	0.072
Diabetic duration (months)	60 (10, 120)	60 (12, 120)	48 (10, 120)	0.247
**BMI (kg/m^2^)**	25.81±4.00	27.07±3.65	23.98±3.80	<0.001^***^
**Waist (cm)**	92.5±11.22	95.27±10.98	87.34±9.85	<0.001^***^
SBP (mmHg)	127 (118, 135)	128 (119, 134)	123 (115, 136)	0.200
DBP (mmHg)	81 (75, 85)	82 (76, 86)	79 (74, 84)	0.138
FPG (mmol/L)	6.86 (5.38, 8.73)	6.94 (5.71, 9.26)	6.79 (5.21, 8.43)	0.129
PPG (mmol/L)	11.04 (8.92, 14.38)	11.21 (9.11, 14.29)	10.98 (8.53, 14.41)	0.381
HbA1c (%)	9.00 (7.40, 11.30)	9.05 (7.40, 11.53)	9.00 (7.30, 10.80)	0.478
TC (mmol/L)	4.96 (4.06, 5.72)	4.93 (4.11, 5.97)	5.04 (4.06, 5.55)	0.678
**TG (mmol/L)**	1.74 (1.15, 2.66)	1.99 (1.31, 3.01)	1.33 (0.95, 2.11)	0.001^**^
LDL (mmol/L)	3.22±0.90	3.20±0.86	3.26±0.96	0.593
**HDL (mmol/L)**	1.04 (0.91, 1.21)	1.02 (0.88, 1.18)	1.07 (0.97, 1.27)	0.027^*^
Cr (μmol/L)	66.80 (56.30, 79.95)	65.23 (55.48, 79.87)	69.55 (59.89, 79.95)	0.191
**UA (μmol/L)**	390.46±107.99	410.30±108.32	361.38±101.17	<0.001^***^
eGFR (mL/min/1.73 m^2^)	101.17 (87.88, 113.65)	102.07 (88.32, 115.01)	97.32 (86.53, 111.28)	0.179
UACR (mg/g)	12.08 (5.35, 49.81)	10.98 (5.43, 48.30)	13.20 (5.29, 60.74)	0.838
ALB (g/L)	39.74 ± 3.38	39.75 ± 2.89	39.71 ± 4.00	0.927
AST (U/L)	20 (16, 28)	22 (16, 30)	18 (16, 25)	0.056
ALT (U/L)	25 (18, 34)	26 (18, 38)	24 (17, 29)	0.065
**FIB-4**	1.14 (0.68, 1.60)	1.26 (0.76, 1.65)	1.03 (0.57, 1.43)	<0.001^***^
**Ewing’s score**	1.00 (0.50, 2.00)	1.00 (0.50, 2.00)	1.00 (0.00, 1.50)	0.001^**^
DPN, n (%)	85 (34.14)	48 (32.43)	37 (36.63)	0.492
DR, n (%)	43 (17.27)	26 (17.57)	17 (16.83)	0.880
Hypertension, n (%)	103 (41.37)	62 (41.89)	41 (40.59)	0.838
**Dyslipidemia, n (%)**	161 (64.66)	105 (70.95)	56 (55.45)	0.012^*^
**Hyperuricemia, n (%)**	104 (41.77)	71 (47.97)	33 (32.67)	0.016^*^
**Metabolic syndrome, n (%)**	185 (74.30)	121 (81.76)	64 (63.37)	0.001^**^
**Obesity, n (%)**	73 (29.32)	59 (39.86)	14 (13.86)	<0.001^***^
**FIB-4>1.3, n (%)**	100 (40.16)	70 (47.30)	30 (29.70)	0.005^**^
**DCAN+, n (%)**	76 (30.52)	54 (36.49)	22 (21.78)	0.013^*^

Continuous data are shown as mean ± standard deviation or median(Q1, Q3), and percentage(%). BMI, body mass index; SBP, systolic blood pressure; DBP, diastolic blood pressure; FPG, fasting plasma glucose; PPG, postprandial plasma glucose; TC, total cholesterol; TG, triglycerides; LDL, low-density lipoprotein; HDL, high-density lipoprotein; Cr, creatinine; UA, urid acid; eGFR, estimated glomerular filtration rate; UACR, urinary albumin-to-creatinine ratio; ALB, albumin; AST, aspartate aminotransferase; ALT, alanine aminotransferase; FIB-4, fibrosisindex-4; DPN, diabetic peripheral neuropathy; DR, diabetic retinopathy; DCAN, diabetic cardiovascular autonomic neuropathy. *P value <0.05, **P value <0.01,***P value <0.001. Bold formatting, statistically significant results.

A comparative analysis of the demographic characteristics of the MAFLD and non-MAFLD groups is presented in **Table 1**. Patients with MAFLD had a longer diabetes duration than non-MAFLD patients, while the HbA1C level did not differ significantly between the two groups (*P*>0.05). T2DM patients with MAFLD had a higher BMI and waist circumference, further supporting the presence of abdominal obesity (*P* < 0.001). These patients also had higher rates of metabolic abnormalities, including dyslipidemia, hyperuricemia, and metabolic syndrome (*P* < 0.05). Dyslipidemia in MAFLD patients was characterized by higher triglyceride and lower high-density lipoprotein levels. The FIB-4 index was higher in the MAFLD group compared with that in the non-MAFLD patients (*P* < 0.001). In addition, high FIB-4 levels (FIB-4>1.3) were more prevalent in the MAFLD group (*P* = 0.005).

**Figure 2:**
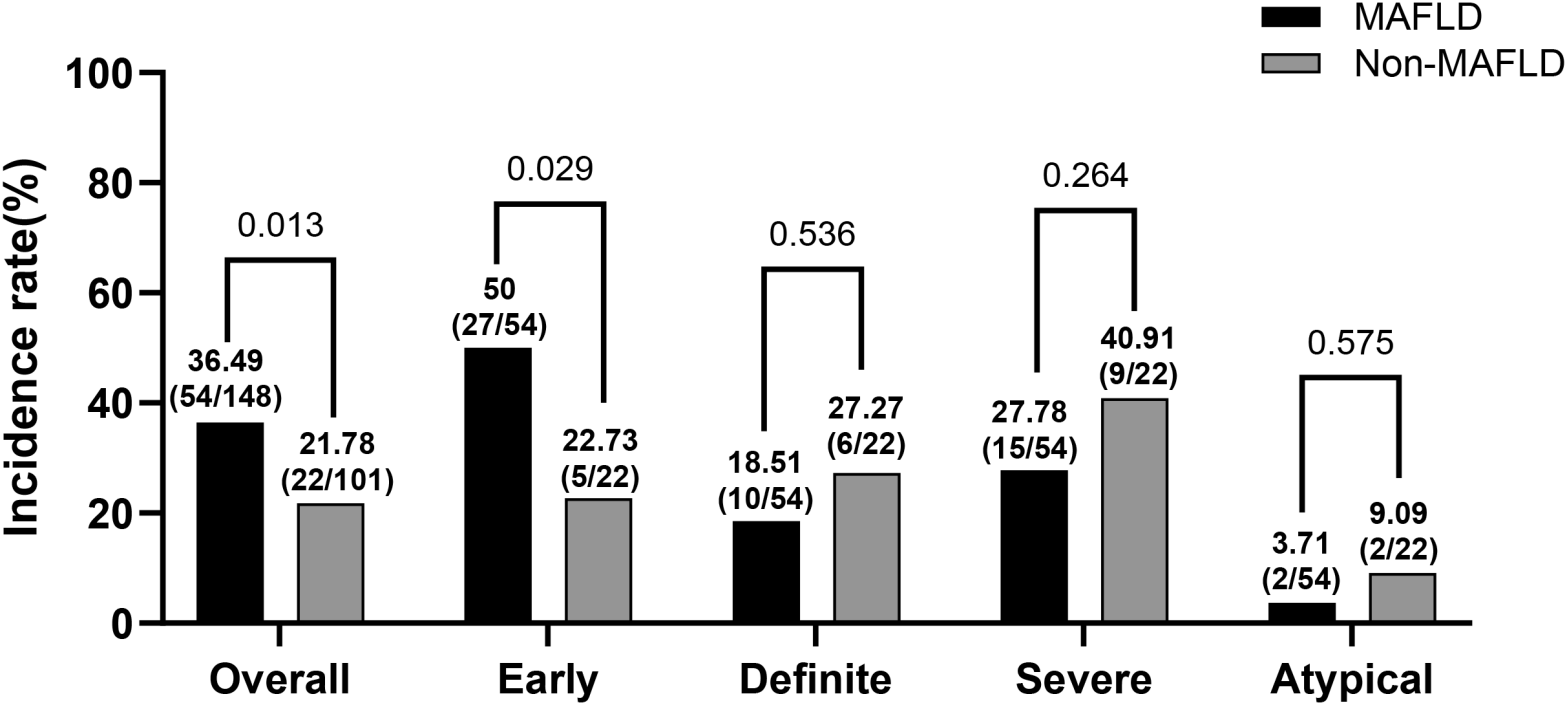
Distribution of cardiovascular autonomic neuropathy in T2DM people with or without MAFLD.

Among the 249 participants, the incidence of DCAN was higher in the MAFLD group than in the non-MAFLD group (36.49% vs. 21.78%, *P* = 0.013). In addition, early DCAN differed significantly between the two groups (*P* = 0.029). In contrast, no significant differences were observed in definite, severe, or atypical DCAN (*P*>0.05) (**Figure 2**).

### 3.2 Stratification characteristics by DCAN presence

Patients with DCAN were older, had longer durations of diabetes, had higher SBP, DBP, and UACR, worse renal function, and a higher prevalence of DPN, DR, and metabolic syndrome than those without DCAN (*P* < 0.05) (**Supplementary Table 3**).

In addition, individuals with DCAN had more MAFLD (71.05% vs. 54.34%, *P* = 0.013), and their FIB-4 levels were significantly higher in participants with DCAN (*P* = 0.004). Additionally, more patients with DCAN presented with high FIB-4 levels (FIB-4>1.3) (47.37% vs. 36.99%, *P* = 0.124).

### 3.3 Relationship between MAFLD and DCAN

The association between clinical variables and diabetic cardiovascular autonomic neuropathy (DCAN) was also examined. Univariable logistic regression analysis revealed that DCAN was significantly associated with age, diabetic duration, SBP, UACR, DPN, DR, hypertension, metabolic syndrome, MAFLD, and the level of FIB-4 (**Table 2**). Notably, the association between MAFLD and DCAN remained significant in the multivariable model after adjustment for factors identified as significant in the univariate logistic analyses: age, sex, diabetes duration, hypertension, DPN, DR, metabolic syndrome, and UACR (adjusted OR = 2.76, 95% CI: 1.44-5.29, *P=0.002*) (**Table 3**).

**Table 2:** Univariate logistic regression analysis of risk factors for diabetic cardiovascular neuropathy.

	OR, 95% CI	*P* value
Male, n (%)	0.84 (0.48 ~ 1.46)	0.525
Age (years)	1.05 (1.02 ~ 1.07)	<0.001^***^
Smoking, n (%)	1.35 (0.76 ~ 2.42)	0.305
Drinking, n (%)	0.80 (0.35 ~ 1.80)	0.581
Diabetic duration (months)	1.01 (1.01 ~ 1.01)	<0.001^***^
BMI (kg/m^2^)	0.98 (0.91 ~ 1.05)	0.521
Waist (cm)	1.00 (0.98 ~ 1.03)	0.721
SBP (mmHg)	1.03 (1.01 ~ 1.05)	0.005^**^
DBP (mmHg)	1.02 (0.99 ~ 1.06)	0.123
FPG (mmol/L)	0.99 (0.89 ~ 1.09)	0.831
PPG (mmol/L)	0.95 (0.88 ~ 1.03)	0.189
HbA1c (%)	1.03 (0.93 ~ 1.15)	0.570
TC (mmol/L)	1.01 (0.87 ~ 1.18)	0.889
TG (mmol/L)	1.07 (0.99 ~ 1.15)	0.105
LDL (mmol/L)	0.74 (0.54 ~ 1.01)	0.058
HDL (mmol/L)	0.86 (0.41 ~ 1.78)	0.677
Cr (μmol/L)	1.00 (0.99 ~ 1.01)	0.988
UA (μmol/L)	1.00 (1.00 ~ 1.00)	0.521
eGFR (mL/min/1.73 m^2^)	0.99 (0.98 ~ 1.00)	0.061
UACR (mg/g)	1.01 (1.01 ~ 1.01)	0.007^**^
ALB (g/L)	0.97 (0.89 ~ 1.05)	0.406
AST (U/L)	0.99 (0.97 ~ 1.01)	0.316
ALT (U/L)	0.99 (0.98 ~ 1.00)	0.158
FIB-4	1.53 (1.07 ~ 2.19)	0.019^*^
DPN, n (%)	3.43 (1.95 ~ 6.05)	<0.001^***^
DR, n (%)	2.62 (1.34 ~ 5.14)	0.005^**^
Hypertension, n (%)	2.45 (1.41 ~ 4.26)	0.001^**^
Dyslipidemia, n (%)	0.61 (0.35 ~ 1.06)	0.078
Hyperuricemia, n (%)	1.02 (0.59 ~ 1.76)	0.943
Metabolic syndrome, n (%)	2.29 (1.14 ~ 4.60)	0.020^*^
Obesity, n (%)	0.89 (0.49 ~ 1.62)	0.699
MAFLD, n (%)	2.06 (1.16 ~ 3.68)	0.014^*^
FIB-4>1.3, n (%)	1.53 (0.89 ~ 2.65)	0.125

BMI, body mass index; SBP, systolic blood pressure; DBP, diastolic blood pressure; FPG, fasting plasma glucose; PPG, postprandial plasma glucose; TC, total cholesterol; TG, triglycerides; LDL, low-density lipoprotein; HDL, high-density lipoprotein; Cr, creatinine; UA, urid acid; eGFR, estimated glomerular filtration rate; UACR, urinary albumin-to-creatinine ratio; ALB, albumin; AST, aspartate aminotransferase; ALT, alanine aminotransferase; FIB-4, fibrosis index-4; DPN, diabetic peripheral neuropathy; DR, diabetic retinopathy; MAFLD, metabolic dysfunction-associated fatty liver disease. *P value <0.05, **P value <0.01,***P value <0.001.

**Table 3:** Association between MAFLD and diabetic cardiovascular neuropathy among T2DM population.

	Unadjusted model		Model 1		Model 2		Model 3	
	OR, 95% Cl	*P* value	OR, 95% Cl	*P* value	OR, 95% Cl	*P* value	OR, 95% Cl	*P* value
MAFLD, n (%)	2.06	0.014^*^	2.36	0.005^**^	2.58	0.003^**^	2.76	0.002^**^
	(1.16, 3.68)		(1.29, 4.31)		(1.38, 4.80)		(1.44, 5.29)	
FIB-4>1.3, n (%)	1.53	0.125	1.07	0.816	1.20	0.567	1.29	0.432
	(0.89, 2.65)		(0.59, 1.94)		(0.65, 2.20)		(0.68, 2.46)	
FIB-4	1.53	0.019^*^	1.17	0.521	1.22	0.442	1.29	0.027^*^
	(1.07, 2.19)		(0.73, 1.88)		(0.74, 2.01)		(1.08, 2.15)	

OR, Odds Ratio, CI, Confidence Interval.

*P value <0.05, **P value <0.01,***P value <0.001. Model 1: adjusted by age, sex. Model 2: adjusted by model 1 plus diabetic duration. Model 3: adjusted by model 2 plus hypertension, diabetic peripheral neuropathy, diabetic retinopathy, metabolic syndrome and UACR.

Subsequently, we further probed the relationship between FIB-4 and DCAN in patients with T2DM stratified by MAFLD status. Among patients with MAFLD, a higher level of FIB-4 (>1.3) was significantly associated with DCAN after fully adjustment for confounding factors (adjusted OR = 2.81, 95% CI: 1.19-6.63, *P=0.018*). In contrast, this association was not observed in patients without MAFLD (**Table 4**).

**Table 4:** ORs and 95% CIs for DCAN according to MAFLD with or without FIB-4>1.3 among T2DM population.

Multivariate models (OR, 95% Cl)	MAFLD (-) (n=101)		MAFLD (+) (n=148)	
	FIB-4 ≤ 1.3 (n=71)	FIB-4 > 1.3 (n=30)	FIB-4 ≤ 1.3 (n=78)	FIB-4 > 1.3 (n=70)
Unadjusted model	Reference(1.00)	1.12 (0.38, 2.17)	Reference (1.00)	2.74 (1.37, 5.47)
		*P* = 0.512		*P=*0.004^**^
Model 1	Reference(1.00)	0.67 (0.33, 2.29)	Reference (1.00)	2.00 (0.95, 4.22)
		*P* = 0.497		*P* = 0.070
Model 2	Reference(1.00)	1.55 (0.22, 4.36)	Reference (1.00)	2.35 (1.08, 5.11)
		*P* = 0.371		*P* = 0.032^*^
Model 3	Reference(1.00)	2.19 (0.42, 6.73)	Reference (1.00)	2.81 (1.19, 6.63)
		*P* = 0.382		*P* = 0.018^*^

OR, Odds Ratio, CI, Confidence Interval.

*P value <0.05, **P value <0.01,***P value <0.001. Model 1: adjusted by age, sex. Model 2: adjusted by model 1 plus diabetic duration. Model 3: adjusted by model 2 plus hypertension, diabetic peripheral neuropathy, diabetic retinopathy, metabolic syndrome and UACR.

To further explore the relationship between MAFLD and DCAN, subgroup analyses were performed based on various baseline characteristics, as demonstrated in **Supplementary Table 4**. The independent positive relationships between the MAFLD and DCAN remained consistent across various subgroups, with no significant interaction effects were detected.

## 4 Discussion

Our study demonstrated that the overall DCAN prevalence was 30.52% among T2DM patients, while 36.49% T2DM patients with MAFLD had DCAN. After multivariable adjustment, MAFLD is significantly associated with DCAN among T2DM patients (adjusted OR = 2.76, 95% CI: 1.44-5.29, *P=0.002*). Critically, among MAFLD patients, an elevated FIB-4 index (>1.3), indicating a higher risk for potential liver fibrosis, was strongly and independently associated with a substantially elevated risk of DCAN.

Owing to the atypicality of DCAN symptoms in its early stages, most patients with T2DM fail to recognize their clinical significance, allowing the condition to progress to severe, uncontrolled outcomes. Therefore, early detection, timely intervention, and risk factor modification may slow or reverse the progression of DCAN.

Several risk factors for DCAN have been identified. *Okdahl* et al. reported that lower high-density lipoprotein levels and higher cardiovascular risk scores were associated with the presence of DCAN (38). *Liu* et al. identified a strong positive relationship between higher HOMA2-IR and the prevalence of DCAN, suggesting its potential utility for DCAN screening (36). Previous studies have also highlighted several metabolic risk indicators, which are associated with DCAN in T2DM patients. Another study revealed that metabolic dysfunction-associated steatotic liver disease was associated with an increased prevalence of atherosclerotic cardiovascular disease in patients with type 1 diabetes mellitus (25). However, the relationship between MAFLD and DCAN has not been thoroughly investigated.

The independent association between MAFLD and DCAN highlights role of MAFLD in a multisystem disorders associated with poor metabolic and cardiovascular poor outcomes (39, 40). Emerging evidence supports a link between hepatic steatosis and cardiac autonomic dysfunction. *Targher* et al. reported that individuals with T2DM and NAFLD alone had an increased risk of cardiac sympathetic and parasympathetic imbalances (20). While previous studies have linked MAFLD to increased cardiovascular risk and hepatic steatosis to impaired cardiac autonomic balance, our study explicitly quantified MAFLD’s impact on DCAN risk in T2DM, rigorously accounting for shared confounders (16, 41).

MAFLD contributes to pathophysiological mechanisms that exacerbate autonomic nerve damage. Several interconnected pathways are likely to mediate this association. Hyperglycemia and obesity not only play crucial roles in the occurrence and development of MAFLD but are also central to the pathogenesis of diabetic neuropathy (42). Insulin resistance impairs neuronal glucose utilization, whereas multiple inflammatory cytokines promote nerve fiber damage and microvascular dysfunction (43). MAFLD, especially in its more advanced histological forms (metabolic steatohepatitis and advanced fibrosis), may exert adverse effects mainly through the systemic release of multiple proinflammatory, prooxidant, and profibrotic mediators, thus contributing to the development of various extrahepatic complications, including functional and structural cardiac abnormalities. Furthermore, MAFLD progression is associated with increased oxidative stress and the release of hepatokines, such as fetuin A and retinol-binding protein 4, which can disrupt systemic metabolic homeostasis and vascular integrity (44). A retrospective longitudinal study demonstrated that high serum CRP level may be a reliable biomarker for predicting the future risk of hospitalization for heart failure in patients with MAFLD (45). These mechanisms likely act synergistically to accelerate autonomic neuropathy in individuals susceptible to diabetes.

The robust association between an elevated FIB-4 level (>1.3), a validated marker indicating the potential risk of fibrosis, and a dramatically higher DCAN risk, specifically within the MAFLD group, is also a pivotal finding. This suggests that the pathophysiological burden associated with the potential risk of fibrosis, rather than steatosis alone, substantially increases DCAN susceptibility. An elevated FIB-4 reflects more advanced hepatic metabolic derangement, potentially associated with amplified systemic inflammation, endothelial dysfunction, and oxidative stress, which may disproportionately impact the autonomic nerves (46). This highlights the value of the FIB-4 index as a practical risk-stratification tool for identifying DCAN among patients with MAFLD.

However, these study had several limitations. Due to the nature of this cross-sectional study, causality between MAFLD and DCAN could not verified. Future large-scale longitudinal studies are needed to further validate these findings. In addition, owing to the lack of fiber-scan data in our study, consistent with previous studies, we defined the FIB-4>1.3 as a high FIB-4 level, indicating a potential risk of liver fibrosis. The association between MAFLD and DCAN was limited by its reliance on FIB-4 as a single non-invasive tool for assessing liver fibrosis. We recommend that future studies validate this association and clarify its underlying mechanisms in dedicated cohorts with fibrosis confirmed by elastography or liver biopsy. Additionally, the long-term effects of some medications, which might affect the diagnosis of DCAN, could not be immediately eliminated after discontinuation; therefore, medications were not included as confounders in the analysis. Failure to adjust for medication use and other potential unmeasured confounding factors (such as detailed lifestyle factors and environmental exposures) represents another important limitation, which may affect the precise estimation of the association strength between MAFLD and DCAN. Furthermore, subgroup analyses by some variables (such as drinking, hyperuricemia, and metabolic syndrome) were compromised by uneven sample sizes, precluding robust assessment of dependent effects on the MAFLD and CAN association. These results require validation using cross-sectional samples with balanced stratification.

## 5 Conclusion

DCAN is highly prevalent in T2DM patients with MAFLD. MAFLD is also independently associated with an increased prevalence of DCAN in patients with T2DM. Critically, among patients with MAFLD, an elevated FIB-4 level (>1.3), signifying a potential risk of liver fibrosis, is associated with a markedly increased risk of DCAN. These findings position MAFLD, particularly elevated FIB-4 levels suggestive of fibrosis progression, as a significant risk indicator for CAN in patients with diabetes. Incorporating MAFLD screening and FIB-4 assessment into clinical practice could help identify T2DM patients at a heightened risk of DCAN, intensifying monitoring and management focused on both metabolic liver disease and cardiovascular autonomic health.

## Data Availability

The raw data supporting the conclusions of this article will be made available by the authors, without undue reservation.

## Supplementary material

Supplementary Table 1 Scoring system of each cardiovascular autonomic reflex tests. Abbreviations: E/I test, deep breathing test; 30:15 test, lying-to-standing (systolic pressure change) test; Handgrip test, diastolic pressure change

Supplementary Table 2 Comparison of baseline characteristics between analytic population and excluded patients due to missing imaging or laboratory data.Continuous data are shown as mean ± standard deviation or median(Q1, Q3), and percentage(%). DCAN, diabetic cardiovascular autonomic neuropathy; BMI, body mass index; SBP, systolic blood pressure; DBP, diastolic blood pressure; FPG, fasting plasma glucose; PPG, postprandial plasma glucose; TC, total cholesterol; TG, triglycerides; LDL, low-density lipoprotein; HDL, high-density lipoprotein; Cr, creatinine; UA, urid acid; eGFR, estimated glomerular filtration rate; UACR, urinary albumin-to-creatinine ratio; ALB, albumin; AST, aspartate aminotransferase; ALT, alanine aminotransferase; DPN, diabetic peripheral neuropathy; DR, diabetic retinopathy.**P* value <0.05, ***P* value <0.01*,***P* value <0.001.

Supplementary Table 3 Cross-sectional sample stratified by the incidence of DCAN.Continuous data are shown as mean ± standard deviation or median(Q1, Q3), and percentage(%). DCAN, diabetic cardiovascular autonomic neuropathy; BMI, body mass index; SBP, systolic blood pressure; DBP, diastolic blood pressure; FPG, fasting plasma glucose; PPG, postprandial plasma glucose; TC, total cholesterol; TG, triglycerides; LDL, low-density lipoprotein; HDL, high-density lipoprotein; Cr, creatinine; UA, urid acid; eGFR, estimated glomerular filtration rate; UACR, urinary albumin-to-creatinine ratio; ALB, albumin; AST, aspartate aminotransferase; ALT, alanine aminotransferase; FIB-4, fibrosis index-4; DPN, diabetic peripheral neuropathy; DR, diabetic retinopathy; MAFLD, metabolic dysfunction-associated fatty liver disease. **P* value <0.05, ***P* value <0.01*,***P* value <0.001.

Supplementary Table 4 Association between MAFLD and diabetic cardiovascular autonomic neuropathy (DCAN) according to baseline characteristics. OR: Odds Ratio, CI: Confidence Interval UA, urid acid; eGFR, estimated glomerular filtration rate; UAER, urinary albumin excretion ratio; DPN, diabetic peripheral neuropathy; DR, diabetic retinopathy.
