# Supplementary material for: Association between metabolic dysfunction-associated fatty liver disease and cardiovascular autonomic neuropathy in type 2 diabetes
Source: Front Endocrinol (Lausanne). 2026 Jan 28;16:1711660. doi: 10.3389/fendo.2025.1711660 (PMC12890623; doi:10.3389/fendo.2025.1711660)
Supplement: Supplementary Table 1 — Scoring system of each cardiovascular autonomic reflex tests. Abbreviations: E/I test, deep breathing test; 30:15 test, lying-to-standing (systolic pressure change) test; Handgrip test, diastolic pressure change [file Table1.docx]

| Test | Normal | Borderline | Abnormal |
| --- | --- | --- | --- |
| Reflecting the function of parasympathetic nervous system | | | |
| Valsalva ratio | ≥1.21 | 1.11-1.20 | ≤1.10 |
| E/I test (beats/min) | ≥15 | 11-14 | ≤10 |
| 30:15 test | ≥1.04 | 1.01-1.03 | ≤1.00 |
| Reflecting the function of sympathetic nervous system | | | |
| Orthostatic hypotension test (mmHg) | ≤10 | 11-29 | ≥30 |
| Handgrip test (mmHg) | ≥16 | 11-15 | ≤10 |

**Supplementary Table 1. Scoring system of each cardiovascular autonomic reflex tests.**

Abbreviations: E/I test, deep breathing test; 30:15 test, lying-to-standing (systolic pressure change) test; Handgrip test, diastolic pressure change

**Supplementary Table 2. Comparison of baseline characteristics between analytic population and excluded patients due to missing imaging or laboratory data.**

|  | Analytic population(n=249) | Excluded Patients(n=218) | *P* value |
| --- | --- | --- | --- |
|  |  |  |  |
| Male, n (%) | 158 (63.45) | 123 (56.42) | 0.121 |
| Age (years) | 53 (41, 61) | 50 (36, 59) | 0.146 |
| Smoking, n (%) | 74 (29.72) | 43 (23.63) | 0.160 |
| Drinking, n (%) | 34 (13.65) | 31 (17.22) | 0.309 |
| Diabetic duration (months) | 60 (10, 120) | 78 (11, 144) | 0.600 |
| BMI (kg/m^2^) | 25.81±4.00 | 26.12 ± 5.36 | 0.776 |
| Waist (cm) | 92.5±11.22 | 90.21 ± 16.72 | 0.053 |
| SBP (mmHg) | 127 (118, 135) | 125 (115, 135) | 0.094 |
| DBP (mmHg) | 81 (75, 85) | 79 (73, 85) | 0.299 |
| **FPG (mmol/L)** | 6.86 (5.38, 8.73) | 5.56 (4.59, 7.33) | <0.001 |
| **PPG (mmol/L)** | 11.04 (8.92, 14.38) | 9.34 (6.84, 12.12) | <0.001 |
| **HbA1c (%)** | 9.00 (7.40, 11.30) | 7.30 (5.80, 9.10) | <0.001 |
| TC (mmol/L) | 4.96 (4.06, 5.72) | 4.92 (3.76, 5.67) | 0.172 |
| TG (mmol/L) | 1.74 (1.15, 2.66) | 1.59 (1.05, 2.18) | 0.078 |
| LDL (mmol/L) | 3.22±0.90 | 2.99 ± 1.05 | 0.184 |
| HDL (mmol/L) | 1.04 (0.91, 1.21) | 1.10 (0.98, 1.29) | 0.141 |
| Cr (μmol/L) | 66.80 (56.30, 79.95) | 69.50 (57.74, 85.00) | 0.081 |
| UA (μmol/L) | 390.46±107.99 | 383.41 ± 106.54 | 0.516 |
| eGFR (mL/min/1.73 m^2^) | 101.17 (87.88, 113.65) | 101.43 (86.39, 111.67) | 0.673 |
| UACR (mg/g) | 12.08 (5.35, 49.81) | 9.19 (5.46, 31.12) | 0.372 |
| ALB (g/L) | 39.74 ± 3.38 | 41.14 ± 3.62 | 0.126 |
| AST (U/L) | 20 (16, 28) | 19 (15, 26) | 0.153 |
| ALT (U/L) | 25 (18, 34) | 20 (13, 34) | 0.244 |
| **DPN, n (%)** | 85 (34.14) | 22 (22.22) | 0.030 |
| DR, n (%) | 43 (17.27) | 21 (20.79) | 0.440 |

Continuous data are shown as mean±standard deviation or median(Q1, Q3), and percentage(%). DCAN, diabetic cardiovascular autonomic neuropathy; BMI, body mass index; SBP, systolic blood pressure; DBP, diastolic blood pressure; FPG, fasting plasma glucose; PPG, postprandial plasma glucose; TC, total cholesterol; TG, triglycerides; LDL, low-density lipoprotein; HDL, high-density lipoprotein; Cr, creatinine; UA, urid acid; eGFR, estimated glomerular filtration rate; UACR, urinary albumin-to-creatinine ratio; ALB, albumin; AST, aspartate aminotransferase; ALT, alanine aminotransferase; DPN, diabetic peripheral neuropathy; DR, diabetic retinopathy.**P* value <0.05, ***P* value <0.01*,***P* value <0.001*.*

**Supplementary Table 3. Cross-sectional sample stratified by the incidence of DCAN.**

|  | DCAN+ (n = 76) | DCAN- (n = 173) | *P* value |
| --- | --- | --- | --- |
|  |  |  |  |
| Male, n (%) | 46 (60.53) | 112 (64.74) | 0.525 |
| Age (years) | 58 (50, 64) | 50 (39, 59) | <0.001^***^ |
| Smoking, n (%) | 26 (34.21) | 48 (27.75) | 0.304 |
| Drinking, n (%) | 9 (11.84) | 25 (14.45) | 0.581 |
| Diabetic duration (months) | 90 (12, 180) | 36 (10, 120) | 0.001^**^ |
| BMI (kg/m^2^) | 25.57 ± 3.71 | 25.92 ± 4.13 | 0.522 |
| Waist (cm) | 92.43 ± 10.70 | 91.88 ± 11.46 | 0.722 |
| SBP (mmHg) | 131 (123, 140) | 125 (117, 133) | 0.003^**^ |
| DBP (mmHg) | 84 (76, 87) | 79 (74, 84) | 0.019^*^ |
| FPG (mmol/L) | 7.03 (5.35, 8.45) | 6.84 (5.41, 8.83) | 0.977 |
| PPG (mmol/L) | 10.53 (8.74, 13.90) | 11.58 (9.09, 14.39) | 0.226 |
| HbA1c (%) | 9.30 (7.75, 10.93) | 8.80 (7.20, 11.40) | 0.378 |
| TC (mmol/L) | 4.92 (3.76, 5.57) | 5.01 (4.19, 5.91) | 0.200 |
| TG (mmol/L) | 1.60 (1.08, 2.66) | 1.81 (1.17, 2.66) | 0.767 |
| LDL (mmol/L) | 3.06 ± 0.77 | 3.29 ± 0.94 | 0.057 |
| HDL (mmol/L) | 1.03 (0.90, 1.21) | 1.04 (0.92, 1.21) | 0.630 |
| Cr (μmol/L) | 66.72 (60.09, 81.11) | 67.00 (56.26, 79.92) | 0.674 |
| UA (μmol/L) | 397.09 ± 101.15 | 387.55 ± 111.02 | 0.522 |
| eGFR (mL/min/1.73 m^2^) | 92.27 (84.93, 104.67) | 102.42 (88.99, 115.50) | 0.009 |
| UACR (mg/g) | 30.36 (8.81, 78.59) | 9.94 (5.03, 24.09) | <0.001^***^ |
| ALB (g/L) | 39.47 ± 3.36 | 39.85 ± 3.39 | 0.407 |
| AST (U/L) | 20 (16, 26) | 21 (16, 28) | 0.573 |
| ALT (U/L) | 22 (16, 28) | 27 (18, 35) | 0.050 |
| FIB-4 | 1.28 (0.99, 1.70) | 1.09 (0.70, 1.44) | 0.004^**^ |
| Ewing’s score | 2.50 (2.00, 3.12) | 0.50 (0.00, 1.00) | <0.001^***^ |
| DPN, n (%) | 41 (53.95) | 44 (25.43) | <0.001^***^ |
| DR, n (%) | 21 (27.63) | 22 (12.72) | 0.004^**^ |
| Hypertension, n (%) | 43 (56.58) | 60 (34.68) | 0.001^**^ |
| Dyslipidemia, n (%) | 43 (56.58) | 118 (68.21) | 0.077 |
| Hyperuricemia, n (%) | 32 (42.11) | 72 (41.62) | 0.943 |
| Metabolic syndrome, n (%) | 64 (84.21) | 121 (69.94) | 0.018^*^ |
| Obesity, n (%) | 21 (27.63) | 52 (30.06) | 0.699 |
| MAFLD, n (%) | 54 (71.05) | 94 (54.34) | 0.013^*^ |
| FIB-4>1.3, n (%) | 36 (47.37) | 64 (36.99) | 0.124 |

Continuous data are shown as mean±standard deviation or median(Q1, Q3), and percentage(%). DCAN, diabetic cardiovascular autonomic neuropathy; BMI, body mass index; SBP, systolic blood pressure; DBP, diastolic blood pressure; FPG, fasting plasma glucose; PPG, postprandial plasma glucose; TC, total cholesterol; TG, triglycerides; LDL, low-density lipoprotein; HDL, high-density lipoprotein; Cr, creatinine; UA, urid acid; eGFR, estimated glomerular filtration rate; UACR, urinary albumin-to-creatinine ratio; ALB, albumin; AST, aspartate aminotransferase; ALT, alanine aminotransferase; FIB-4, fibrosis index-4; DPN, diabetic peripheral neuropathy; DR, diabetic retinopathy; MAFLD, metabolic dysfunction-associated fatty liver disease. **P* value <0.05, ***P* value <0.01*,***P* value <0.001*.*

| **Subgroup** | **OR (95% CI)** | ***P value*** | **P for interaction** |
| --- | --- | --- | --- |
| All patients | 2.76 (1.44, 5.29) | 0.002 |  |
| Sex |  |  | 0.672 |
| Male | 2.96 (1.27, 6.91) | 0.012 |  |
| Female | 2.08 (0.70, 6.17) | 0.188 |  |
| Age (years) |  |  | 0.247 |
| ≤60 | 2.07 (0.99, 4.33) | 0.055 |  |
| >60 | 9.07 (1.88, 43.77) | 0.006 |  |
| Diabetic duration (months) |  |  | 0.150 |
| ≤120 | 4.15 (1.62, 10.6) | 0.003 |  |
| >120 | 1.43 (0.48, 4.23) | 0.517 |  |
| Smoking |  |  | 0.061 |
| No | 1.92 (0.85, 4.34) | 0.119 |  |
| Yes | 7.17 (1.82, 28.15) | 0.005 |  |
| Hypertension |  |  | 0.319 |
| No | 3.34 (1.27, 8.79) | 0.014 |  |
| Yes | 2.13 (0.79, 5.76) | 0.136 |  |
| Dyslipidemia |  |  | 0.387 |
| No | 3.70 (1.29, 10.67) | 0.015 |  |
| Yes | 2.18 (0.88, 5.41) | 0.093 |  |
| Obesity |  |  | 0.268 |
| No | 2.37 (1.16, 4.87) | 0.018 |  |
| Yes | 21.32 (1.19, 383.14) | 0.038 |  |
| UA (μmol/L) |  |  | 0.662 |
| ≤420 | 3.53 (1.55, 8.02) | 0.003 |  |
| >420 | 1.76 (0.48, 6.48) | 0.395 |  |
| eGFR (mL/min/1.73m²) |  |  | 0.418 |
| ≥90 | 3.15 (1.48, 6.72) | 0.003 |  |
| <90 | 0.85 (0.16, 4.37) | 0.844 |  |
| UAER (mg/24h) |  |  | 0.123 |
| ≤30 | 3.64 (1.50, 8.82) | 0.004 |  |
| >30 | 1.10 (0.35, 3.51) | 0.870 |  |
| DPN |  |  | 0.360 |
| No | 2.10 (0.89, 4.94) | 0.089 |  |
| Yes | 4.70 (1.64, 13.47) | 0.004 |  |
| DR |  |  | 0.936 |
| No | 2.98 (1.40, 6.36) | 0.005 |  |
| Yes | 3.33 (0.73, 15.10) | 0.119 |  |

**Supplementary Table 4. Association between MAFLD and diabetic cardiovascular autonomic neuropathy (DCAN) according to baseline characteristics.**

OR: Odds Ratio, CI: Confidence Interval

UA, urid acid; eGFR, estimated glomerular filtration rate; UAER, urinary albumin excretion ratio; DPN, diabetic peripheral neuropathy; DR, diabetic retinopathy.
